# The Emergence, Persistence, and Dissemination of Antimicrobial-Resistant Bacteria in Environmental Hajj Settings and Implications for Public Health

**DOI:** 10.3390/tropicalmed6010033

**Published:** 2021-03-10

**Authors:** Jaffar A. Al-Tawfiq, Ziad A. Memish

**Affiliations:** 1 Specialty Internal Medicine and Quality Division, Johns Hopkins Aramco Healthcare, Dhahran 31311, Saudi Arabia; jaffar.tawfiq@jhah.com; 2 Infectious Disease Division, Department of Medicine, Indiana University School of Medicine, Indianapolis, IN 46202, USA; 3 Infectious Disease Division, Department of Medicine, Johns Hopkins University School of Medicine, Baltimore, MD 21093, USA; 4 Director Research and Innovation Center, King Saud Medical City, Ministry of Health, Riyadh 12746, Saudi Arabia; 5 Al-Faisal University, Riyadh 11533, Saudi Arabia; 6 Hubert Department of Global Health, Rollins School of Public Health, Emory University, Atlanta, GA 30322, USA

**Keywords:** Hajj, mass gathering, antimicrobial resistance

## Abstract

The emergence of antimicrobial resistance is causing the loss of what was once considered the miracle cure. The transmission of antimicrobial resistance during mass gathering is a potential threat in addition to other infectious diseases. Here, we review the English language literature on the rate and the acquisition of antimicrobial resistance during the Hajj. There is a variable incidence of methicillin-resistant *Staphylococcus aureus*, *Escherichia coli*, and Enterobacteriaceae. There had been no report of multi-drug-resistant *Mycobacterium tuberculosis*. Continued surveillance of antimicrobial resistance coupled with public health measures are needed to decrease the rate of emergence of resistance.

## 1. Introduction

It is known that there is an increasing trend in antimicrobial resistance among Gram-negative and Gram-positive bacteria. In a systematic review, the prevalence of findings, the prevalence of vancomycin-resistant *Enterococcus faecium* is high in the Eastern Mediterranean countries [1]. Another study showed increasing antimicrobial resistance among *Acinetobacter baumannii* globally as well [2]. The issue of antimicrobial resistance might be even exaggerated in the setting of mass gatherings such as the annual Hajj. There is also an increase in antimicrobial-resistant bacteria in the Kingdom of Saudi Arabia [3].

**Figure 1:**
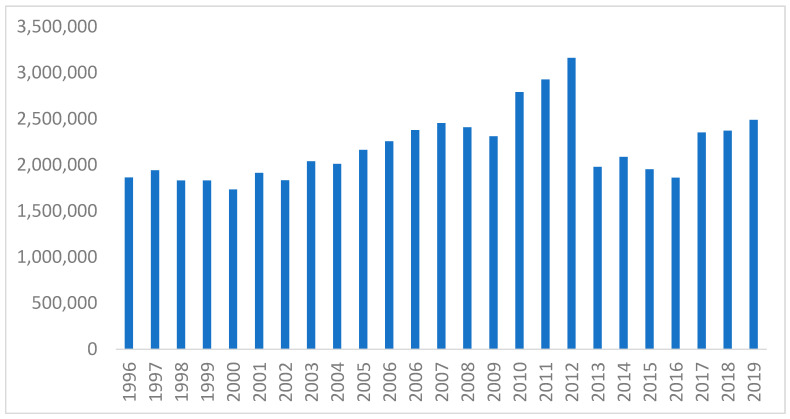
The number of the annual Hajj pilgrims per year 1996–2019.

The annual Muslim Pilgrimage, Hajj, and the mini-Hajj, Umrah, are recurrent mass gathering events that take place annually in Makkah, Kingdom of Saudi Arabia (KSA). The annual Hajj is one of the largest recurrent mass gatherings (MGs) and accommodates two to three million Muslims who come from > 180 countries around the world [4,5]. This annual Hajj is considered one of five pillars of Islam and is a mandatory to be performed by able and capable adult Muslims once in a lifetime. However, Umrah is a shorter version of the Hajj and can be done optionally at any time during the year, whereas the Hajj has to be done in a specified period time in relation to the Lunar, Islamic, Calendar [5,6,7]. The estimated annual pilgrimage for both Hajj and Umrah is more than 10 million pilgrims [8]. The number of the annual Hajj pilgrims per year has increased from 1996 till 2012 when the grand mosque expansion project was started (Figure 1). The number of pilgrims decreased significantly during the COVID-19 pandemic.

The Kingdom of Saudi Arabia, through its Ministry of Health, has provided curative and preventative medical services to all pilgrims through the establishment of several hospitals and clinics in the Hajj premises. One of its many mandates is to control any potential spread of infectious diseases during the Hajj [8,9,10,11,12]. Prior to each Hajj season, the Saudi Ministry of Health coordinates with pertinent health authorities in the countries of origin of the pilgrims to develop suitable material for education and update on Hajj medical requirements [9].

Mass gathering events are associated with increased risk of transmission of infectious diseases especially respiratory tract infections [5,7,13,14,15,16]. Multiple reasons had been identified as risk factors for such occurrences and include attendance of a large number of pilgrims in congested overcrowded areas among many others. The repeated emergence of novel viral pathogens have always been feared with the recent emergence of pH1N1 in 2009, Middle East Respiratory Syndrome Coronavirus (MERS-CoV) in 2012, and the Severe Acute Respiratory Syndrome Coronavirus 2 (SARS-CoV-2) in 2020 [8,10,16,17,18,19].

The occurrence of diarrheal illnesses among attendees of mass gatherings is well established and continues to be included in the surveillance program during the Hajj [9,12,20,21,22]. The reported rates of diarrhea among pilgrims range from 1.1% to 23.3% in multiple cohort studies of 262,999 pilgrims from 2002 to 2013 [20].

Diarrheal illness among Hajj pilgrimage is another area of concern as it might be caused by antimicrobial-resistant organisms with the potential to spread during the mass gathering event. In one study, the most frequently associated pathogens in patients with diarrhea during the Hajj were: *Salmonella* spp., *Shigella*, enteroinvasive *Escherichia coli* (EIEC) and enterotoxigenic *Escherichia coli* (ETEC) [23]. In a systematic review of 15 studies of 262,999 pilgrims from 2002 to 2013 showed that the prevalence of diarrheal illness among pilgrimage was 2.3% (range: 1.1–23.3%) [21]. Cited risk factors for diarrhea among pilgrimage were: eating in restaurants [24] and being male [25,26], however, the source of food and eating raw vegetables were not associated significantly with the occurrence of diarrhea [25]. Diarrheal illness was reported among 12% of attendees in the annual Arbaeen mass gatherings in Iraq [27] and among 4.5% among attendees of the 2015 Grand Magal [28] in one study, and 14.5% had gastrointestinal symptoms in another study [29].

## 2. Antimicrobial-Resistant Bacteria among Diarrheal Pathogens

In one study, 40% of *Salmonella* and *E. coli*-diarrheal pathogens were extended-spectrum β-lactamases (ESBLs) and carbapenemases such as *bla*_CTX-M-15_ and *bla*_NDM_ elements [23]. This high rate is alarming giving the fact that the Hajj pilgrimage attracts > 2 million pilgrims from 188 countries [22]. In another study, 2 of 267 rectal swabs were positive for *Salmonella* Newport serotype and these were resistant to multiple antibiotics (cephalosporins, gentamicin and colistin) and had *bla(CTX-M-2)* gene and had colistin resistance [30].

## 3. Antimicrobial-Resistant Bacteria among Enterobacteriaceae

Resistance to third generation cephalosporins was reported among 19–94% of *E. coli* and K. [31,32,33,34]. Extended spectrum beta-lactamase (ESBL)-producing Enterobacteriaceae was found among pilgrims and multiple isolates had CTX-M type ESBL genes [30,35]. Another study of urinary *E. coli* isolates in pilgrims showed the presence of *E. coli* ST131 and ST648 [36]. One study of French pilgrims showed one *E. coli* isolate with *blaNDM-5*, *blaCTX-M-15*, *blaTEM-1*, and *aad*A2 (ST2659 and ST181) genes [37]. The presence of the mcr-1 plasmid mediating colistin resistance was found at a rate of 9% [38]. ESBL genes, *blaCTX-M*, *blaTEM*, and *blaSHV*, were reported in two studies conducted in ICU patients. The proportion of *blaCTX-M* and *blaTEM* in *E. coli* and *K. pneumoniae* cases was 18.5–30%, *blaSHV* was 7.4% and 17.2% among *E. coli* and *K. pneumoniae*, respectively [39,40]. The rate of imipenem-resistance among *E. coli* and *K. pneumoniae* was 4–11.9% [31,34,41]. For carbapenem-resistant *P. aeruginosa* isolated from patients, 4.1–18.4% carried *blaVIM* and 4.7–21.0% carried *blaIMP* [39,42,43]. Shiga toxin-producing *E. coli* among diarrheal isolates showed 70% resistance to trimethoprim-sulfamethoxazole [44]. A number of studies showed different extended-spectrum-β-lactamase (*bla*) genes that had been identified in the Hajj [23,35,36,37,45,46] (Table 1).

## 4. Antimicrobial Resistance among *Acinetobacter baumannii* and *P. aeruginosa*

The rate of imipenem-resistance among *A. baumannii* and *P. aeruginosa* was 4–60.5% and 4–43%, respectively. The prevalence of *bla*_OXA-23_ was 91% among *A. baumannii* causing infection in ICU patients [39]. The occurrence of metallo-β-lactamase genes among carbapenem-resistant *A. baumannii* isolates was 11.5–27.1% for *bla*_VIM_ and 13.6% for *bla*_IMP._ [39,42,43]. In a study of French pilgrims, the rate of isolation of ceftriaxone-resistant *A. baumannii* with *bla*_OXA-51-like_ gene was 14.4% throat, 25.6% rectal, and 3.3% from throat and rectal swabs [37]. The study was showed one *A. baumannii* isolate with imipenem resistance and had *bla*_OXA-72_ carbapenemase gene [37].

## 5. Occurrence of Methicillin-Resistant *Staphylococcus aureus* (MRSA) and Vancomycin-Resistant *S. aureus* (VRSA)

The incidence of methicillin-resistant Staphylococcus aureus (MRSA) in isolates from patients during the Hajj had steadily increased overtime in parallel with the incidence in the community and the healthcare settings [47]. MRSA incidence was 2–7% in pilgrims in 2000–2004 [48], 28% in patients with sinusitis in 2014 [49] and 63% in community acquired infections in 2015 [50]. The rate of MRSA was 15–20% in Hajj pilgrimages and 10–11% in Umrah pilgrims and the rate was similar before and after participating in the events [51]. The incidence of MRSA among food handlers in Makkah was 0% in 2001–2002 and was 20% in 2014 [52,53]. Occurrence of vancomycin-resistant *S. aureus* (VRSA) was 2% among pilgrims [54]. The specific resistance gene was investigated among *S. aureus* isolates and these studies showed the presence of Panton-Valentine leucocidin (PVL) in 0–19% [55,56]. The presence of the fibronectin binding protein (fnBPA)-encoding gene in MRSA was 8% [55]. A summary of studies examining the occurrence of Methicillin-Resistant Staphylococcus aureus in the Hajj pilgrims and workers is shown in Table 2 [33,41,48,49,50,51,52,53,54,55,56,57,58].

## 6. Resistance among Other Gram-Positive Organisms

Resistance to vancomycin among *Enterococcus faecalis* was 3.5% and 2% for *Enterococci* [54]. Oxacillin-resistant coagulase-negative staphylococci (CoNS) rate was 61% in 2004–2005, 82.4% in 2008–2009, and 93.6% in 2012–2013 [41,54,59].

## 7. Occurrence of Antibiotic-Resistant Respiratory Bacterial Pathogens among Pilgrimages

In a study of the carriage of *Streptococcus pneumoniae* among pilgrims showed that 23% of such isolates were resistant to multiple antibiotics (≥3 classes of antibiotics) [60] with a rate of 21% for ampicillin-resistant *S. pneumoniae* [54]. In the same cohort study among 110 isolates from pilgrims, *S. pneumoniae* resistance was as follows: 30.9% were penicillin non-susceptible, 2.7% were intermediately resistant to amoxicillin and 1.8% were intermediately resistant to cefotaxime, 24.5% were resistant to erythromycin, 12.7% were resistant to clindamycin, 55.5% were resistant to tetracycline, 6.4% were resistant to chloramphenicol, 48.2% were resistant to trimethoprim-sulfamethoxazole, and 16.4% were intermediately resistant to trimethoprim-sulfamethoxazole [60].

## 8. Tuberculosis and the Hajj

Due to the crowding conditions and the fact that 50% of pilgrims arrive from places with high prevalence of tuberculosis, including Africa, Bangladesh, India, Pakistan and Southeast Asia [61]. The occurrence of pulmonary tuberculosis had been described among pilgrims. However, the data regarding drug resistance are sparse. The prevalence of active pulmonary tuberculosis is variable (1.2–10%) among hospitalized patients [61,62,63,64,65] (Table 3). One study of 1063 pilgrims showed that 15 (1.4%) had pulmonary tuberculosis and there was no multidrug-resistant cases [66]. However, there had been no description of any outbreak of tuberculosis related to the Hajj.

## 9. Transmission of Meningococcal Disease

*Neisseria meningitidis* is one of the most studied organisms when it comes to the Hajj and pilgrimage and it was associated with multiple outbreaks [11,67,68,69,70,71,72,73]. There had been two major outbreaks of *N. meningitides* in relation to the 1987 Hajj and the 2000–2001 [11,67,68,69]. Asymptomatic *N. meningitidis* was >80% and had a significant impact on the Hajj outbreaks during Hajj in 1987 and 2000–2001 [69,74]. In a recent study of 628 paired cohort pilgrims, the rate of acquisition of *N. meningitidis* was 5.7% on arrival and 2.5% on departure [68]. The outbreak in 1987 was caused by *N. meningitidis* serogroup A [74,75,76], and serogroup W135 [77]. The outbreaks in 2000–2001 were also associated with resistance to trimethoprim-sulfamethoxazole, sulfadiazine, cloxacillin and tetracycline [78]. Following the first outbreak the bivalent A and B meningococcal vaccine became mandatory and the quadrivalent (ACYW135) vaccine became mandatory after the second outbreak. The occurrence of azithromycin resistance among pilgrims was 8.3% and 10.3% before and after Hajj pilgrimage and 5% for ciprofloxacin [51]. Another study showed resistance among *N. meningitides* was as follows 5% to ciprofloxacin, 12% to ceftriaxone, 3% to rifampicin, and 9% to azithromycin [79].

## 10. Other Resistant Pathogens

The prevalence of resistance among *Helicobacter pylori* was 31% for metronidazole and 3% for tetracycline and erythromycin [80].

## 11. Discussion

It had been shown that mass gatherings such as the Hajj are significant pools for the spread and transmission of antimicrobial-resistant bacteria due to crowded conditions, droplet transmission, and lack of efficient personal hygiene [21]. There had been an increased rates of AMR in the Hajj pilgrims. The occurrence of plasmid-mediated resistance among bacteria may suggest that these bacteria may be transmitted across different pilgrims. It was shown that the spread of clones and specific AMR genes are associated with travel and food [81]. The detection of these AMR in pilgrims may provide an area for further research and investigation of ways of transmissions especially that the Hajj time changes overtime and falls in the summer season every 10 years [82].

Meningococcal disease continues to be a potential public risk at the Hajj due to the invasiveness of the disease, increasing antimicrobial resistance, diverse serotypes (A, C, W-135, Y, and others), changes in the incidence, and alterations in serogroups and genotypes [83]. Colonization by *N. meningitidis* can be a major potential source of infection and is a potential source of the spread to other parts of the world [9,60,84,85]. There had been several studies examining the rate of *N. meningitidis* and the rate was variable from 0% to 6.3% [86,87,88]. One study of a paired cohort showed a prevalence of *N. meningitidis* of 2.5% on arrival and 1.3% on departure [87,88]. Another paired cohort study showed a prevalence of 2.5% on arrival and 0.15% upon departure to have *N. meningitidis* [68]. A third cohort study showed rates of 0.3% and 0.6% of *N. meningitides* among paired cohort and non-paired cohort, respectively [89]. Another cohort French study showed 0% of *N. meningitides* on arrival and departure [90]. Ciprofloxacin prophylaxis had shown to decrease *N. meningitidis* carriage as follows: no ciprofloxacin group had 5.2% before and 4.6% after pilgrimage (P = 0.65) and with ciprofloxacin the rate was 8.1% and 0% before and after pilgrimage [91]. Mandatory ciprofloxacin prophylaxis is given to pilgrims arriving from Sub-Saharan African meningitis belt countries [10,74,92,93,94].

Use of antimicrobial agents without a prescription is a major drive to the development of antibiotic resistance and this is true in the case of the Hajj [95] and one study showed that 47.6% of pilgrims used antibiotics [96].

The main modes of transmission of antimicrobial-resistant bacteria are respiratory, direct contact, and food-borne. As such it is important to maintain a high level of infection control compliance among pilgrims and healthcare settings [97]. The profession of safe food is also very important. In addition, there is a need for fast and reliable diagnostic tests. The utilization of available vaccines would also contribute to the efforts to decrease transmission of antimicrobial-resistant organisms especially respiratory tract infections. There needs to be ongoing surveillance of bacterial and viral pathogens and the development of antimicrobial resistance among pilgrims utilizing electronic surveillance systems [9,98].

One important method to decrease the risk of antimicrobial-resistant organisms is vaccination that would decrease the risk of infection and thus the use of antimicrobial therapy. The Saudi ministry of health annually revise the recommendations for the utilization of vaccines during the Hajj [6,99]. These vaccines include the required and recommended vaccines. The rate of pneumococcal vaccination among pilgrims had been variable [18,60,89,90,100,101,102,103,104,105,106] (Table 4).

In addition, the Saudi authorities had banned the selling of antibiotics without a prescription. It is also important to continue the surveillance activities for the emergence of any antimicrobial-resistant organisms during the Hajj. The Saudi ministry of health had adopted electronic surveillance system [9]. The surveillance could be staged and may involve multiple methods such as syndromic and laboratory based surveillance [107]. The World Health Organization (WHO) had adopted the 5-4-8-4 approach to reduce the risk of antimicrobial resistance during the Hajj [108] and this could be applied to other mass gathering events as well. This approach included the five pillars of the WHO strategy that includes: increase awareness and surveillance, reduce infections, optimize antimicrobial use, and the development of an economic case for maintainable investment in antimicrobial resistance [109]. The next number, four, refers to the need to have clinicians adhere to the four moments of antibiotic prescribing strategy. This moment includes: the presence of bacterial infection requiring antibiotics, the need to order appropriate cultures and to initiate the appropriate empiric antibiotics, how long should antibiotics be prescribed, and when antibiotics could be stopped [110]. The number eight refers to the priority pathogens: *Acinetobacter* spp., *E. coli*, *K. pneumoniae*, *N. gonorrhoeae*, *Salmonella* spp., *Shigella* spp., *S. aureus*, *S. pneumoniae*. The last number refers to the four specimens to be collected for culture (blood, urine, stool, urethral and cervical swabs) [108].

The risk of tuberculosis among pilgrims is of particular importance. There had been no reported outbreaks of tuberculosis among pilgrims. However, the situation of pilgrims and the overcrowding conditions may promote the spread of respiratory pathogens such as tuberculosis and viral infections such as the Middle East Respiratory Syndrome Coronavirus (MERS-CoV) and the Severe Acute Respiratory Syndrome Coronavirus 2 (SARS-CoV-2). For tuberculosis, it is assuring to note that the recent global burden of tuberculosis study estimates that there is a trend in the decline in the incidence of the disease and if such decline continues then a few countries may meet the Sustainable Development Goal (SDG) target to eliminate tuberculosis by 2030 [111]. The risk of the development of MERS-CoV was a real issue. However, a systematic review in 2018 showed no evidence of MERS-CoV among pilgrims [7]. A subsequent study of 28,197 returning pilgrims to Indonesia showed no evidence of MERS-CoV infection as well [112]. The emergence of SARS-CoV-2 and the subsequent development of the pandemic had attracted lots of attention towards the Hajj. Thus, the Kingdom of Saudi Arabia had taken multiple steps to prevent the introduction of the virus to the pilgrims. It was expected that the Hajj season in 2020 would be cancelled due to the pandemic [113]. Actually, Saudi Arabia suspended the Umrah (mini-Hajj) and limited the access to the 2020 Hajj [8]. With continued pandemic and decreasing cases in Saudi Arabia, the government had taken steps to ease the strictions and developed a staged approach to scaling up the Umrah Pilgrimage in the last few months of 2020 [114].

To control the increasing antimicrobial resistance globally, two important public health proposals are to include education and training of the medical students during undergraduate and post-graduate studies. For example, in one study, 81% of 1055 young physicians indicated that antimicrobial resistance was not adequately addressed during medical training [115]. This is further exemplified by a study of the knowledge, attitude and practice of healthcare workers deployed during two Hajj seasons, where 85% indicated hearing about antimicrobial resistance and 19% had heard about antimicrobial stewardship programs [116]. In addition, it is important to include education and training of the young physicians on the discipline of mass gathering to alert them to the medical risks and challenges [117].

## Figures and Tables

**Table 1:** A Summary of the different extended-spectrum-β-lactamase (*bla*) genes that had been identified in the Hajj.

Gene	Organism	Reference
*blaCTX-M-15*	*Salmonella* spp. and *E. coli*	[23,36,37]
*blaOXA-72*	*Acinetobacter baumannii*	[37]
*blaNDM-5*	*Escherichia coli*	[37]
*blaCTX-M*	*Escherichia coli*, *K. pneumoniae*	[35,36,37,45]
*blaNDM*	*Salmonella*, enterotoxigenic *E. coli*, *K. pneumoniae*,	[23,37,46]
*blaOXA*	*E. coli*, *K. pneumoniae*, *Acinetobacter baumannii*	[36,37,46]
*blaTEM*	*E. coli*, *K. pneumoniae,*	[35,36,37]
*blaSHV*	*E. coli*, *K. pneumoniae,*	[35,36]
*blaVIM*	*K. pneumoniae*	[46]
*aac*6, *aac*6Ib, *aad*A4, *str*B, *aad*A1, *aad*A2, *aad*B, *ant*2, *aph*A, *str*A	aminoglycoside-resistant *E. coli*	[36]

**Table 2:** A summary of Studies Examining Methicillin-Resistant *Staphylococcus aureus* in the Hajj pilgrims and workers.

Study Year	Source	Number of Individuals with MRSA/No. of Individuals with Positive Culture (%)	Reference
2000–2001	Skin lesion	1/47 (2.1)	[48]
2000–2001	Multiple sites	0/45 (0)	[52]
2004	Nasal, axilla, groin and open wound swabs	6/85 (7.1)	[57]
2009	Nasal swabs	16/155 (10.3)	[51]
2009	Nasal swabs	30/153 (19.6)	[51]
2014	Nasal and hand skin swabs	33/165 (20.0)	[53]
2014	Sinus	13/46 (28.3)	[49]
2015	Urine, blood, sputum	36/57 (63.2)	[50]
2003–2004	Wound swabs, ear swabs, eye swabs, blood, urine, respiratory tract	199/512 (38.9)	[58]
2004–2005	Blood	161/303 (53.0)	[41]
2008–2009	Multiple	271/688 (39.4)	[54]
2011–2012	Foot infection and urinary tract infection samples	15/26 (57.7)	[33]
2012	No data	100/206 (48.5) mec 19/100 (19.0) PVL	[56]
No data	Blood cultures, wound swabs, urine, nasal swabs, and sputum	11/50 (22.0)	[55]

**Table 3:** A summary of studies describing active tuberculosis among Pilgrims.

Number of Admitted Pilgrims	Number (%) with Active Tuberculosis	Reference
30	3 (10)	[62]
46	9 (20)	[63]
808	10 (1.2)	[64]
452	22 (4.9)	[61]
118	1 (1)	[65]
1063	15 (1.4)	[66]

**Table 4:** Pneumococcal vaccination rates in Hajj pilgrims.

Year of Pilgrimage	Prevalence of Pneumococcal Vaccination (%)	Reference
2011–2013	14.2–28.7	[101]
2009	31.4	[102]
2010	1.7	[103]
2012	35.9	[104]
2013	51.2	[90]
2004–2005	2.5–8.9	[105]
2005	5	[106]
2013	4.4	[18]
2013	1.4	[60]
2013	1.2	[89]
2011–2012	11.3	[100]
